# Chitosan Gel to Treat Pressure Ulcers: A Clinical Pilot Study

**DOI:** 10.3390/pharmaceutics10010015

**Published:** 2018-01-17

**Authors:** Virginia Campani, Eliana Pagnozzi, Ilaria Mataro, Laura Mayol, Alessandra Perna, Floriana D’Urso, Antonietta Carillo, Maria Cammarota, Maria Chiara Maiuri, Giuseppe De Rosa

**Affiliations:** 1 Department of Pharmacy, Università degli Studi di Napoli Federico II, Via D. Montesano 49, 80131 Naples, Italy; virginia.campani@unina.it (V.C.); laumayol@unina.it (L.M.); 2 M.D. Department of Plastic and Reconstructive Surgery and Burn Unit, Hospital Hospital “A. Cardarelli”, Via A. Cardarelli 9, 80131 Naples, Italy; elianapagnozzi@virgilio.it (E.P.); ilariamataro@gmail.com (I.M.); 3 First Division of Nephrology, Department of Cardio-thoracic and Respiratory Sciences, Second University of Naples, School of Medicine, via Pansini 5, Ed. 17, 80131 Naples, Italy; alessandra.perna@unicampania.it; 4 U.O.S.C Farmacia, U.O.S.S. Galenica Clinica e Preparazione Farmaci Antiblastici, Hospital “A. Cardarelli”, Via A. Cardarelli 9, 80131 Naples, Italy; florianad-urso@hotmail.it (F.D.); antoniettacarillo1985@gmail.com (A.C.); maresas@tin.it (M.C.); 5 U.M.R.S. 1138, Centre de Recherche des Cordeliers, 15, rue de l’Ecole de Médecine, 75006 Paris, France; chiara.maiuri@crc.jussieu.fr

**Keywords:** chitosan, gel, pressure ulcers, chronic wounds, wound healing, clinical study

## Abstract

Chitosan is biopolymer with promising properties in wound healing. Chronic wounds represent a significant burden to both the patient and the medical system. Among chronic wounds, pressure ulcers are one of the most common types of complex wound. The efficacy and the tolerability of chitosan gel formulation, prepared into the hospital pharmacy, in the treatment of pressure ulcers of moderate severity were evaluated. The endpoint of this phase II study was the reduction of the area of the lesion by at least 20% after four weeks of treatment. Thus, 20 adult volunteers with pressure ulcers within predetermined parameters were involved in a 30 days study. Dressing change was performed twice a week at outpatient clinic upon chronic wounds management. In the 90% of patients involved in the study, the treatment was effective, with a reduction of the area of the lesion and wound healing progress. The study demonstrated the efficacy of the gel formulation for treatment of pressure ulcers, also providing a strong reduction of patient management costs.

## 1. Introduction

Pressure ulcers are localized areas of injury to the skin and they mainly affect patients that require bed rest. They are caused by external forces, such as pressure, or shear, or a combination of both, and often occur over bony prominence [1]. Wound resolution is often impaired by bacterial proliferation and the production of exudates that causes maceration of healthy skin layers [2,3,4]. Moreover, many factors like smoking, obesity, old age, and malnutrition can promote the development of chronic skin damage and impair healings processes [3]. Wound care represents a heavy cost on the total health care budget [5]. Pressure ulcers have been shown to increase length of hospital stay and associated hospital costs. Costs are mainly dominated by health professional time and, for more severe ulcers, by the incidence of complications, including hospital admission/length of stay [6]. Advanced wound dressings have prohibitive costs for public health system. The economic care impacts of wound healing represent a serious bottleneck for the correct wound care, especially in public hospitals.

Chitosan (CHI) is a natural polysaccharide that is composed of units of glucasamine linked by a 1–4 glycosid bond to N-acetyl glucosamine units [7]. Due to its characteristics of biodegradability, biocompatibility and safety, CHI has attracted considerable interest for biological applications. The presence of a positive charge at physiological pH makes CHI adhesive, ensuring a longer permanence in the application site [8,9]. Furthermore, the antiseptic activity of CHI was also demonstrated [10]. Finally, its abundance in nature and the low-cost of production make this polymer of commercial interest and suitable to be used for a large-scale production [11]. Many studies have demonstrated the effect of CHI in wound healing due to its microbiological activity, and to the ability to promote homeostasis and angiogenesis processes [12,13,14,15,16]. Moreover, CHI positive charges attract growth factors that enhance cell growth and proliferation [17]. In particular, severe infiltrations of polymorphonuclear cells and thick scab have been reported when treating skin wound with CHI-based dressings in dogs [18]. Recently, our research group reported an experimental protocol to prepare the CHI gel suitable for a hospital pharmacy [19]. These CHI-based gels demonstrated the ability to promote wound healing in vitro and in vivo in an animal model of pressure ulcer [19].

Here, we report a pilot clinical study on 20 patients with pressure ulcers and treated with the CHI gels prepared into the hospital. In this study, the efficacy and the tolerability of the treatment were evaluated. The aim of study was to provide a proof-of-concept to support further study on this device, prepared with low-cost biomaterial and directly into the hospital, to reduce the management cost of hospitalized patients affected by pressure ulcers.

## 2. Materials and Methods

Chitosan from crab shells, highly viscous (>400 mPa·s 1% acetic acid at 20 °C) was purchased from Farmalabor (Canosa di Puglia, Italy), acetic acid was obtained by Carlo Erba (Milano, Italy), sterile water was purchased from B. Braun (Milan, Italy), regenerated cellulose 0.22 microM membranes were obtained by Corning (Viesbaden, Germany), and the immediate sterile packaging was kindly offered by Alfamed (Naples, Italy).

### 2.1. Gel Preparation

**Figure 1:**
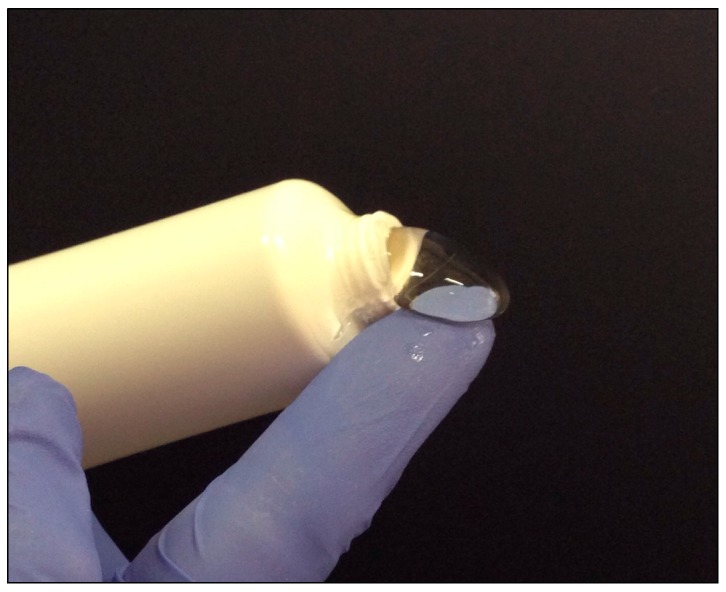
Chitosan gel formulation used in the study.

CHI gels were prepared at the Unità di Manipolazione di Chemioterapici Antiblastici (U.M.A.C.A.) center situated in the Azienda di rilievo nazionale, A.O.R.N. Antonio Cardarelli (Naples, Italy). Gels were prepared, as previously described by Mayol et al. [19] with same modifications. Briefly, CHI powder was sterilized in autoclave at 121 °C for 20 min and 2 atm. Sterilization was checked by microbiological tests carried out on CHI samples at the Laboratorio Chimico Merceologico (Naples, Italy). Samples preparation was made under laminar flow hood and directly in the immediate sterile packaging. The acetic acid aqueous solution was filtered on 0.22 microM membrane filters before use. Then, 0.1 M acetic acid solution was slowly added under continuous stirring to 2% CHI powder until the obtainment of a clear solution. To evaporate the organic solvent, gels were sealed with 0.22 microM filter caps and then placed in oven for 48 h at 37 °C under vacuum (Vuototest, Mazzali, Monza, Italy); finally, filters were removed and the samples were sealed with hermetic caps. Each formulation was prepared in 30 mL sterile container (kindly provided by Alfamed s.r.l., Naples, Italy), intended for a single administration and stored at 4 °C (Figure 1).

### 2.2. Patients Eligibility

Volunteers patients of both sexes, aged between 40 to 80 years with good nutrition conditions, a life expectancy of at least six months with the ability to sign informed consent and affected by pressure ulcers of moderate severity (class II EUPAP/NPUAP 2014) were enrolled. The study excluded subject with: age less than 40 years or older than 80 years, malnutrition state, predisposition to bleeding, or treatment with anticoagulants, infections (including HIV positive), infected injuries, patients not available to follow the procedures envisaged by the study.

A total of 20 adult volunteers with skin ulcers were involved in this 30 days study. Only patients susceptible to outpatient treatment were recruited and hospitalization was not envisaged at any stage of the study. The protocol for the clinical study (identification code: CHITODERM) was examined and approved by ethics committee (No. 558 of 06/24/2016) of “Cardarelli-Santobono” responsible for the experimentation and biomedical research activities carried out at the A.O.R.N. Antonio Cardarelli and A.O.R.N. Santobono-Pausilipon.

### 2.3. Patients Retirement

Patients had the opportunity to retire from the clinical trial at any time and with no obligation to motivate the interruption. Moreover, treatment discontinuation has been provided in case of adverse events such as erythema, itching, and pain. In this case, motivations were attached to the medical record of the patient and no patient replacement was expected. For these patients a follow-up of 30 days duration was planned.

### 2.4. Study Design and Treatment

Pressure ulcers were treated according to the EUPAP/NPUAP guidelines. Firstly, the lesion was cleaned with povidone iodine solution and finally washed with physiological solution. The gel preparation was applied on the decubitus ulcer covering the total area of the lesion. Once filled with the CHI gel, the skin lesion was covered with a secondary dressing. Dressing change was performed two times a week at the outpatient clinic for chronic wound treatment. The study lasted 30 days.

### 2.5. Safety and Efficacy Assessment

The endpoint of the phase II study was the reduction (expressed as a percentage) of the area of the lesion by at least 20% after four weeks of treatment. The secondary endpoint of the study was to establish the tolerability of the gel preparations in the treatment of pressure ulcers. The occurrence of adverse events such as erythema, itching, and pain was evaluated. Patients assessed their degrees of overall satisfaction with the treatment using a 100 mm long horizontal line visual analog scale (VAS).

Patients were treated twice a week. Before each application and at the end of the treatment, the area of the skin lesion was measured. Moreover, any influence of concomitant therapies and the general status of the patient were evaluated.

The area of the lesion was evaluated by digital photography, applying a ruler beside the lesion. Digital images were analyzed using the open source software “Image J” (Java 1.8.0_112) to calculate the area of the wound. At 14 and 30 days visit patient’s satisfaction level was assessed by VAS score, ranging from not satisfied (score-0) to fully satisfied (score-100) with the treatment outcomes.

### 2.6. Statistical Analyses

All of the skin lesion areas were analyzed. The data obtained for each lesion area changes (assessed by image analysis) and presented as area (cm^2^) and as percentage (%) of reduction of the area of lesion were then statistically analyzed. A one-sample Student’s *t*-test was utilized, and the results were analyzed with the statistics software GraphPad Prism Version 6.0a for Macintosh (GraphPad Software, San Diego, CA, USA).

## 3. Results and Discussion

The aim of this trial was to test the efficacy of the gel to accelerate the healing of pressure ulcers. CHI is a biocompatible, biodegradable, and low cost natural polymer proposed for several biopharmaceutical applications, among them wound healing [13,14,15,16]. Although the clinical use of CHI as a wound healing agent has shown difficulty in taking off, its ability to promote tissue regeneration is well known [13,14,15,16]. Different effects, among them inhibition of the microbial growth and increased homeostasis, able to promote healing of injured tissues have been ascribed to CHI. In particular, CHI has been found to be involved in the rapid mobilization of platelets and red blood cells to the injured site and also in vasoconstriction and activation of blood clotting factors responsible for blood clotting [4,12,13]. Thus, CHI accelerates the granulation phase in wound healing and stimulates macrophage activity. Finally, the *N*-acetyl-*D*-glucosamine, which os responsible for fibroblast proliferation, increases collagen and HA synthesis in the wound cavity, and it also allows oxygen permeability at the wound site [12,13]. At the moment, few CHI-based wound dressings (i.e., ChitoFlex^®^, ChitoGauze^®^, ChitoSAM™, Celox™ Rapid Gauze) are available on the market, but proposed for their hemostatic properties although than for tissue regeneration. This pilot clinical study is aimed to support the use of a CHI-based device to increase the wound healing rate, independently on the bleeding of the wound.

Here, to carry out the study, the preparation protocol previously developed [19] was reproduced in a sterile manufacturing area at the U.M.A.C.A. center of the A.O.R.N. Antonio Cardarelli. Thus, in the first phase of the work, three batches prepared into the hospital were characterized in terms of viscoelastic behavior, showing no significant differences with gel previously prepared [19]. Gels were prepared and stored in hermetically sealed containers, to avoid following growth of microbiological contamination. Microbiological tests carried out at the Laboratorio Chimico Merceologico of Naples confirmed that all the three pilot batches prepared in this study were sterile and suitable to be administrated on damaged skin (data not shown).

The clinical protocol was designed according to the 2014 EUPAP/NPUAP guidelines. In particular, following the cleansing of the wound, CHI gel was spread on the skin lesions, then covered with a secondary dressing. The primary endpoint to investigate the efficacy of the treatment was the reduction of the area of the lesion at least 20% after 30 days of treatment of the skin lesion. Indeed, a reduction of the area of the lesion of about 20% observed after the first weeks of treatment can be considered a predictive healing factor [20]. In Table 1, the percentages of reduction of the area of the lesion after 30 days of treatment were reported. As shown, a significant reduction of the area of the lesion (higher than 20%) was observed in most patients (about 90%) with a complete wound healing in 20% of the cases after four-week of treatment with CHI gel. Moreover, in 18 patients the treatment was effective, showing a significantly reduction of the area of the lesion and wound healing progression. Furthermore, in 50% of patients involved in the clinical study, the reduction of the area of the lesion was higher than 50% (patients 1, 2, 5, 6, 7, 9, 10, 14, 16, 17), with a complete wound healing of the ulcers in some cases (patients 2, 5, 9, 16). The results obtained from *t* test demonstrated that the reduction of the area of the lesions after 30 days of treatment was statistically significant with a two-tailed *p* value of 0.0002 (*t* value of 4.16 and a *p* value of 0.0002).

**Figure 2:**
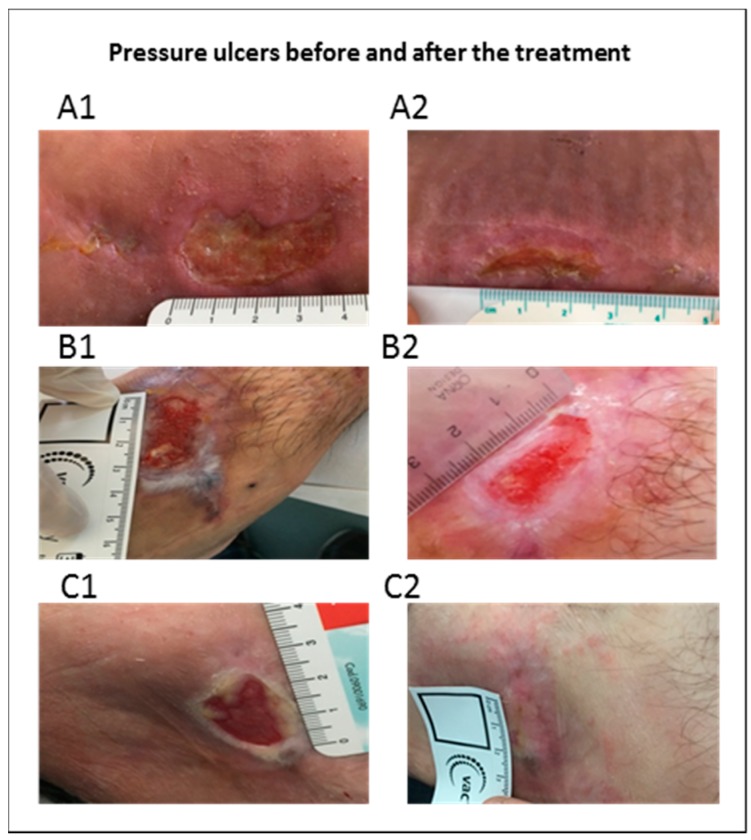
Images of pressure ulcers before (A1, B1, C1) and after 30 days of treatment with chitosan gel (A2, B2, C2).

In Figure 2, representative images of the wounds in three patients before the treatment (panels A1, B1, C1) and after 30 days of treatment (panels A2, B2, C2) are showed. Interestingly, any patient reported adverse effects of mild, moderate of serious severity after the administration of the gel preparations. Finally, in any case, the discontinuation of the treatment was required.

Furthermore, we evaluated the overall patient satisfaction level on a visual analogue scale (VAS) ranging from not satisfied (score-0) to fully satisfied (score-100). The VAS scale was used due to its advantages in the evaluation of satisfaction outcome; indeed, VAS scale is reported as a very powerful research tool, reliable, sensitive, and easy to use [21]. As expected, the patient who obtained a significant reduction of the area of the lesion gave VAS score higher than those patients who did not obtain a significant result after 30 days of treatment (Table 2).

These results on patients confirm our previous findings in an animal model of pressure ulcers [19], where a significant reduction of the area of the lesion after 3 and 10 days of treatment with CHI gel was found. On the other hand, the healing process should not be observed in chronic wound, such as pressure ulcers, during 12 weeks, from the beginning of the treatment [22]. On the contrary, in this study, significant wound healing progression was observed in the majority of the patients (90%) treated with the CHI gel for four weeks. Interestingly, the 50% of the patients the reduction of the area was superior to the 50% compared to the area before the treatment; finally, 20% of the patients resulted completely healed. These encouraging results confirmed the efficacy of the CHI gel in wound healing, suggesting that this formulation could provide real advantages in term of efficacy and the cost of the treatment. A clinical trial on a larger group of patients (phase III) could provide further information on the use of CHI gels on patients with pressure ulcers and other kind of ulcers. Moreover, in this case, only patients with ulcers at the stage II were enrolled. Other studies should also be organized to investigate the effect of CHI gels also on ulcers of a higher severity.

## 4. Conclusions

In conclusion, this pivotal study on a restricted group of subjects affected by pressure ulcers demonstrated the tolerability and the efficacy of the CHI-based gel formulation in promoting wound healing. Although on a limited number of volunteers, 90% of the treated patients were responder to treatment, with 20% of the patients completely healed. Furthermore, in this study the gel was prepared directly into the hospital, in the sterile area of the hospital pharmacy. This approach could represent an alternative to the marketed dressing of innovative biomaterials that are generally quite expensive and not always available in public hospital. On the contrary, CHI gel preparation is very easy to perform and the materials that are used in the preparation have negligible costs. Once that the efficacy of this device will be demonstrated on a larger number of patients (Phase III), these findings could represent the basis of new protocols making together increased healing rate with cost-saving for the public health systems.

## Figures and Tables

**Table 1:** Area of the lesion before and after treatment with chitosan (CHI) formulation and the percentage of reduction of the lesion for each patient.

Patient	Area of the Lesion (before Treatment)	Area of the Lesion (after Treatment)	Reduction of the Area of the Lesion (%)
1	30,238	13,457	55
2	1245	125	90
3	12,580	10,742	15
4	7271	5421	25
5	7356	1031	86
6	7205	3379	53
7	8479	3881	54
8	17,492	9090	48
9	2500	670	73
10	10,832	4329	60
11	2352	1564	34
12	1929	1527	21
13	3687	2901	21
14	2263	146	94
15	33,403	26,336	21
16	33,403	26,336	97
17	1346	460	66
18	14,407	9910	31
19	32,366	20,927	35
20	14,699	13,994	5

**Table 2:** Patient’s satisfaction level assessed by visual analogue scale (VAS) score at 14 days and 30 days visit.

Patient	VAS Score	VAS Score
	14 Days	30 Days
1	41	75
2	63	94
3	19	25
4	34	36
5	74	91
6	45	82
7	56	68
8	44	70
9	55	88
10	70	86
11	42	59
12	28	43
13	31	59
14	75	100
15	15	40
16	66	95
17	63	84
18	27	49
19	36	45
20	15	21

## References

[1] Westby M.J., Dumville J.C., Soares M.O., Stubbs N., Norman G. (2017). Dressings and topical agents for treating pressure ulcers. Cochrane Database Syst. Rev..

[2] Mutsaers S.E., Bishop J.E., McGrouther G., Laurent G.J. (1997). Mechanism of tissue repair: From wound healing to fibrosis. Int. J. Biochem. Cell Biol..

[3] Pereira R.F., Barrias C.C., Granja P.L., Bartolo P.J. (2013). Advanced biofabrication strategies for skin regeneration and repair. Nanomedicine.

[4] Agrawal P., Soni S., Mittal G., Bhatnagar A. (2014). Role of polymeric biomaterials as wound healing agents. Int. J. Low Extrem. Wounds.

[5] Lindholm C., Searle R. (2016). Wound management for the 21st century: Combining effectiveness and efficiency. Int. Wound J..

[6] Dealey C., Posnett J., Walker A. (2012). The cost of pressure ulcers in the United Kingdom. J. Wound Care.

[7] Tomihata K., Ikada Y. (1997). In vitro and in vivo degradation of films of chitin and its deacetylated derivatives. Biomaterials.

[8] He P., Davis S.S., Illum L. (1998). In vitro evaluation of the mucoadhesive properties of chitosan microspheres. Int. J. Pharm..

[9] Calvo P., Remunan-Lopez C., Vila-Jato J.L., Alonso M.J. (1997). Novel chitosan derivatives enhance the transport of hydrophilic hydrophilic chitosan-polyethylene oxide nanoparticles as protein carriers. J. Appl. Polym. Sci..

[10] Burkatovskaya M., Castano A.P., Demidova-Rice T.N., Tegos G.P., Hamblin M.R. (2008). Effect of chitosan acetate bandage on wound healing in infected and noninfected wounds in mice. Wound Repair Regen..

[11] Khor E., Lim L.Y. (2003). Implantable applications of chitin and chitosan. Biomaterials.

[12] Muzzarelli R., Tarsi R., Filippini O., Giovanetti E., Biagini G., Varaldo P.E. (1990). Antimicrobial properties of *N*-carboxybutyl chitosan. Antimicrob. Agents Chemother..

[13] Ueno H., Mori T., Fujinaga T. (2001). Topical formulations and wound healing applications of chitosan. Adv. Drug Deliv. Rev..

[14] Wang W., Lin S., Xiao Y., Huang Y., Tan Y., Cai L., Li X. (2008). Acceleration of diabetic wound healing with chitosan-crosslinked collagen sponge containing recombinant human acidic fibroblastgrowth factor in healing-impaired STZ diabetic rats. Life Sci..

[15] Boateng J.S., Matthews K.H., Stevens H.N., Eccleston G.M. (2008). Wound healing dressings and drug delivery systems: A review. J. Pharm. Sci..

[16] Charernsriwilaiwat N., Rojanarata T., Ngawhirunpat T., Opanasopit P. (2014). Electrospun chitosan/polyvinyl alcohol nanofibre mats for wound healing. Int. Wound J..

[17] Lee D.W., Lim H., Chong H.N., Shim W.S. (2009). Advances in chitosan material and its hybrid derivatives: A review. Open Biomater. J..

[18] Ueno H., Yamada H., Tanaka I., Kaba N., Matsuura M., Okumura M., Kadosawa T., Fujinaga T. (1999). Accelerating effects of chitosan for healing at early phase of experimental open wound in dogs. Biomaterials.

[19] Mayol L., De Stefano D., Campani V., De Falco F., Ferrari E., Cencetti C., Matricardi P.L., Maiuri R., Carnuccio A., Gallo M.C. (2014). Design and characterization of a chitosan physical gel promoting wound healing in mice. J. Mater. Sci. Mater. Med..

[20] Flanagan M. (2003). Improving accuracy of wound measurement in clinical practice. Ostomy Wound Manag..

[21] Singer A.J., Church A.L., Forrestal K., Werblud M., Valentine S.M., Hollander J.E. (1997). Comparison of patient satisfaction and practitioner satisfaction with wound appearance after traumatic wound repair. Acad. Emerg. Med..

[22] Boateng J., Catanzano O. (2015). Advanced Therapeutic Dressings for Effective Wound Healing—A Review. J. Pharm. Sci..

